# Case Report: Extensive Tumor Profiling in Primary Neuroendocrine Breast Cancer Cases as a Role Model for Personalized Treatment in Rare and Aggressive Cancer Types

**DOI:** 10.3389/fmed.2022.841441

**Published:** 2022-06-03

**Authors:** Dörthe Schaffrin-Nabe, Stefan Schuster, Andrea Tannapfel, Rudolf Voigtmann

**Affiliations:** ^1^Praxis für Hämatologie und Onkologie, Bochum, Germany; ^2^Datar Cancer Genetics Europe GmbH, Eckersdorf, Germany; ^3^Institute of Pathology, Ruhr-University Bochum, Bochum, Germany

**Keywords:** extensive tumor profiling, rare cancer therapy, primary endocrine breast cancer, personalized treatment, targeted therapies

## Abstract

Neuroendocrine breast cancer (NEBC) is a rare entity accounting for <0.1% of all breast carcinomas and <0.1% of all neuroendocrine carcinomas. In most cases treatment strategies in NEBC are empirical in absence of prospective trial data on NEBC cohorts. Herein, we present two case reports diagnosed with anaplastic and small cell NEBC. After initial therapies failed, comprehensive tumor profiling was applied, leading to individualized treatment options for both patients. In both patients, targetable alterations of the PI3K/AKT/mTOR pathway were found, including a PIK3CA mutation itself and an STK11 mutation that negatively regulates the mTOR complex. The epicrisis of the two patients exemplifies how to manage rare and difficult to treat cancers and how new diagnostic tools contribute to medical management.

## Introduction

Primary neuroendocrine breast cancer (NEBC) comprises a heterogeneous group of tumors with a low incidence (0.1%) among all breast cancer subtypes (1). In the literature, NEBCs are generally associated with poor long-term survival and with rapid resistance development (1–3). Therapeutic guidelines have not been established to date. The diagnosis of NEBC is often challenging as other neuroendocrine tumors, but also lung, gastrointestinal, and pancreatic cancers, need to be excluded (4, 5).

Currently, surgical intervention is the mainstay of the therapeutic approach (5, 6).

Treatment strategies are chosen dependent on Classification of Malignant Tumors (TNM) status, aggressiveness, age, general condition, and comorbidities of the patient (7). If (neo-)adjuvant chemotherapy is necessary, NEBC is being treated either analog to adenocarcinomas of the breast or SCLC (8, 9). Previously, Ki67 was used as a decision tool in NEBC; Ki67 <15% led to a breast cancer analog therapy, i > 15% of the therapy was orientated to SCLC/neuroendocrine treatment (7). Promising results were seen when a combination of surgery, radiotherapy, and chemotherapy was applied (6).

There are no guidelines for staging and therapy in the metastatic setting, leaving the treating oncologist to opt for suitable systemic treatments (10, 11).

The development of molecular tumor profiling in recent years increasingly provides the opportunity for the use of targeted therapies, taking into account the involved activation and inhibition of the signal transduction pathways (12–14). This tool is particularly useful for rare tumors without existing therapy guidelines and for tumors that are refractory to therapy.

We want to illustrate the diagnostic and therapeutic challenges presenting the epicrisis of two patients diagnosed with NEBC in these above-mentioned situations.

## Patient 1

**Figure 1:**
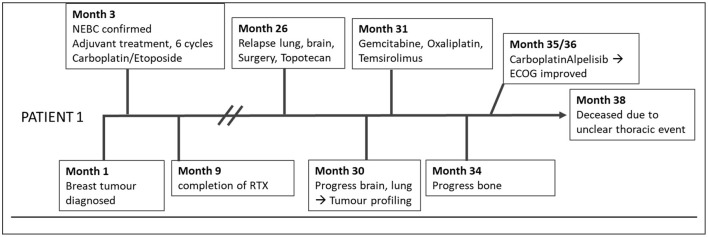
Timeline of patient 1.

The first patient was a 67-year-old female (Figure 1), who had a primary NEBC of the small cell subtype confirmed by histopathology. The definitive tumor stage was pT2, pN1a, L1, V0, G3, and Ki67 at 60%. She underwent modified radical mastectomy with axillary dissection and adjuvant administration of six cycles of Carboplatin and Etoposide, followed by radiotherapy. There was no indication for radiotherapy of the neurocranium as performed in SCLC.

Two years later, pronounced bilateral pleural metastasis without effusion was detected and one brain metastasis on the left occipital side was surgically removed. Both are related to the previously described NEBC. Considering micrometastases of the brain, the patient received Topotecan.

Further brain metastases, progressive lung metastasis with effusion, and metastatic spread to bone and thyroid gland were discovered by MRI 2 months afterward during ongoing chemotherapy.

Consequently, tumor profiling was performed with the exacta^®^ test using peripheral blood to detect genetic alterations by NGS, targetable markers by immunocytochemistry staining, pharmacogenetics of tumor specific medication, and chemotherapy sensitivity testing using circulating tumor associated cells (Table 1) (15–17).

**Table 1:** Main results of the tumor profiling.

	**Case 1**	**Case 2**
Genetic Mutations/Amplifications	PIK3CA p.E545K	TP53 p.R290fs; STK11 p.Y131; NOTCH1 pQ1155; PIK3CG/D; FGFR2 amplification
Pathway Modeling (mRNA based)	Decreased DHFR signaling, increased HIF1 signaling	Increased signaling of TUBB2A, PGF, VEGFA, HIF1
Chemosensitivity Cell Death Rate [%]	Gemcitabine 72%	Gemcitabine +Carboplatin 85%
	Oxaliplatin 59%	Etoposide 79%
	Vinblastine 58%	Gemictabine 60%
	Etoposid <25%	5-Fluoruracil 56%
	Topotecan <25%	Carboplatin 55%
IHC Staining (PD-L1, EGFR, VEGFA, mTOR)	EGFR	-
MMR/MSI	Negative	MSI stable
Tumour Mutational Burden	0, 59 mutations/Mb blood-based	2, 21 mutations/Mb tissue based
Pharmacogenomics (altered metabolism)	ERCC1, NT5C2, UGT1A1, ABCB1	ERCC1, CYP2D6, UGT1A1, FCGR2A

## Patient 2

**Figure 2:**
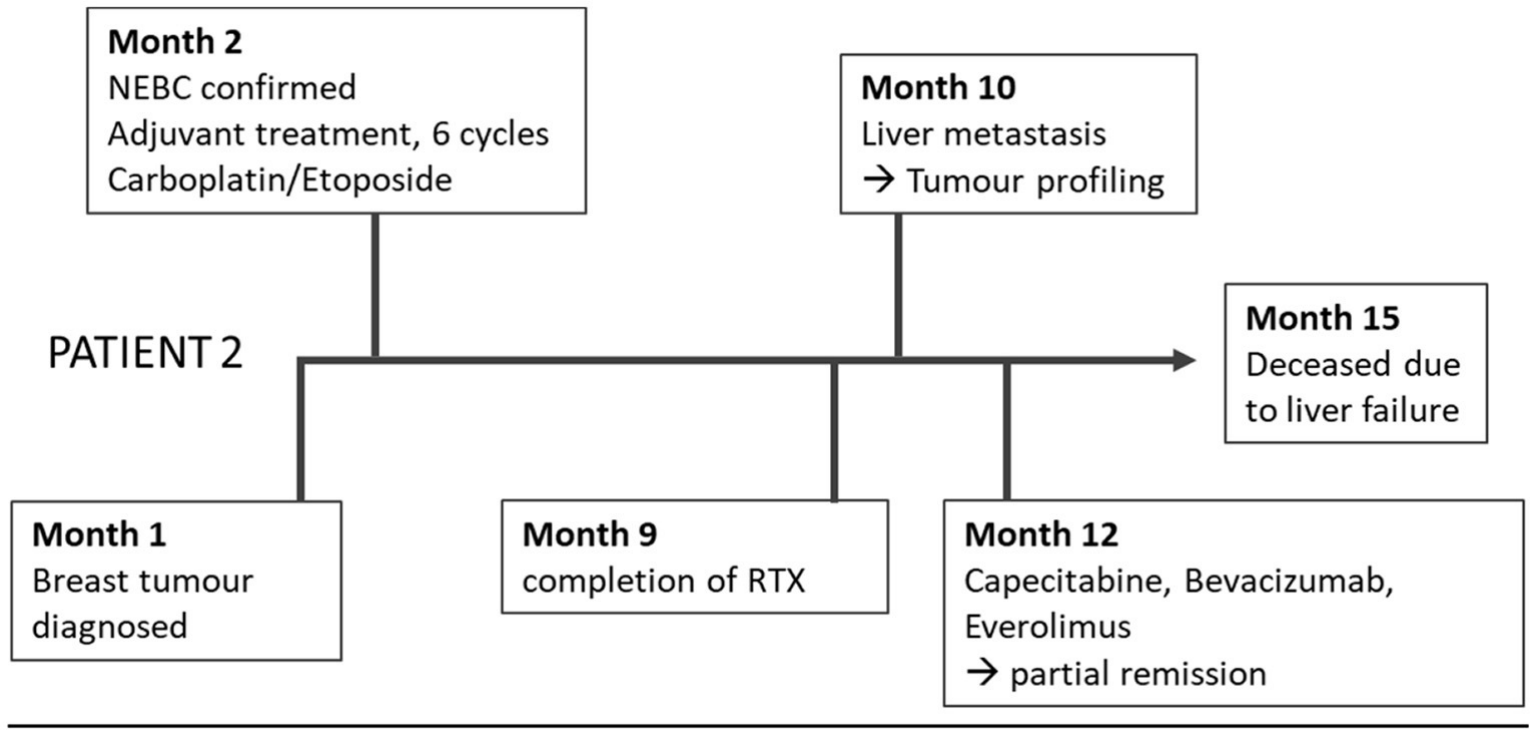
Timeline of Patient 2.

The second 51-year-old female patient suffered from an NEBC anaplastic large cell subtype (Figure 2). After breast-conserving therapy and sentinel lymphonodectomy, the definitive tumor stage was pT2, pN0, G3, Ki 67 40%, L0, V0, Pn0, ER 30%, PR neg, and Her2/neu neg. In the adjuvant setting, carboplatin and etoposide were applied with extremely poor clinical tolerability. Shortly after completion of adjuvant radiotherapy, hepatic filiae appeared in the right liver lobe. The planned atypical liver resection was rejected, because of intraoperatively detected diffuse spread into the left lobe.

Histopathologic confirmation revealed highly proliferating liver metastasis with a Ki67 of 80%, poorly differentiated, associated with the known NEBC.

Tumor profiling was performed using exacta^®^ analysis this time based on a tumor biopsy together with a blood sample (Table 1). Simultaneous to this analysis, a diffuse bone metastasis with infiltration of the spinal canal with corresponding clinical signs was observed and radiotherapy was applied.

## Discussion

The reported aggressive scenario in both patients is consistent with high grading and high proliferation index (Ki67 > 60%). The initial chemotherapies failed and raised the question of novel therapeutic strategies. Dotatate-based PET-CT as an experimental diagnostic and therapeutic alternative was rejected (18). Both cases required the use of newly available diagnostic tools like NGS considering that recommendations for genomic alterations in specific tumor types exist, but not in NEBC or other neuroendocrine tumors (NET). At no stage, guidelines or clinical trials were available, only the individual approach was left.

In the case of the first patient, exacta^®^ revealed an activating mutation of PIK3CA p.E545K, which is one of the most mutated genes and has been found to play a crucial role in several cancer types, but information about the incidence in NEBC is inconsistent in the literature (19–21). The PI3K/AKT/mTOR pathway is highly important for proliferation, migration, and cell survival and alterations are quite frequent in other NETs (22). The mutation, therefore, suggested a therapeutic benefit from mTOR and PIK3CA inhibition. Due to extended metastasis (pleura, neurocranium, and bone) and high Ki67, Gemcitabine and Oxaliplatin were added based on the chemosensitivity result to the mTOR inhibitor Temsirolimus (16, 23). Even though the therapy was tailored to individual tumor characteristics, the patient progressed, developing new pulmonary metastasis and lymphangitis, as well as pronounced pleural effusion. No response was seen despite molecular genetic evidence, together with an upregulation at the messenger RNA (mRNA) level of AKT, an important activator of mTOR, thus, suggesting a potential benefit from mTOR inhibitors. Resistance mechanisms to mTOR inhibitors, for example, caused by disruption of the negative feedback loop between SGK1 and PI3K signaling, followed by AKT activation, could explain treatment failure (24–26). Furthermore, RHEB (RAS homolog enriched in brain) as an mTOR activator was downregulated, together with mTOR downstream activating pathway components, like eIF4B (eukaryotic initiation factor 4B) and S6 (ribosomal subunit S6 = RPS6) (27, 28).

In this case, activation of mTOR seemed to have a lesser impact concerning tumor cell proliferation. Subsequently, she underwent pleurodesis on both sides. TROP2 overexpression relating to Sacituzumab-Govitecan (29, 30), or biomarkers for immune checkpoint inhibitors, were not observed.

**Figure 3:**
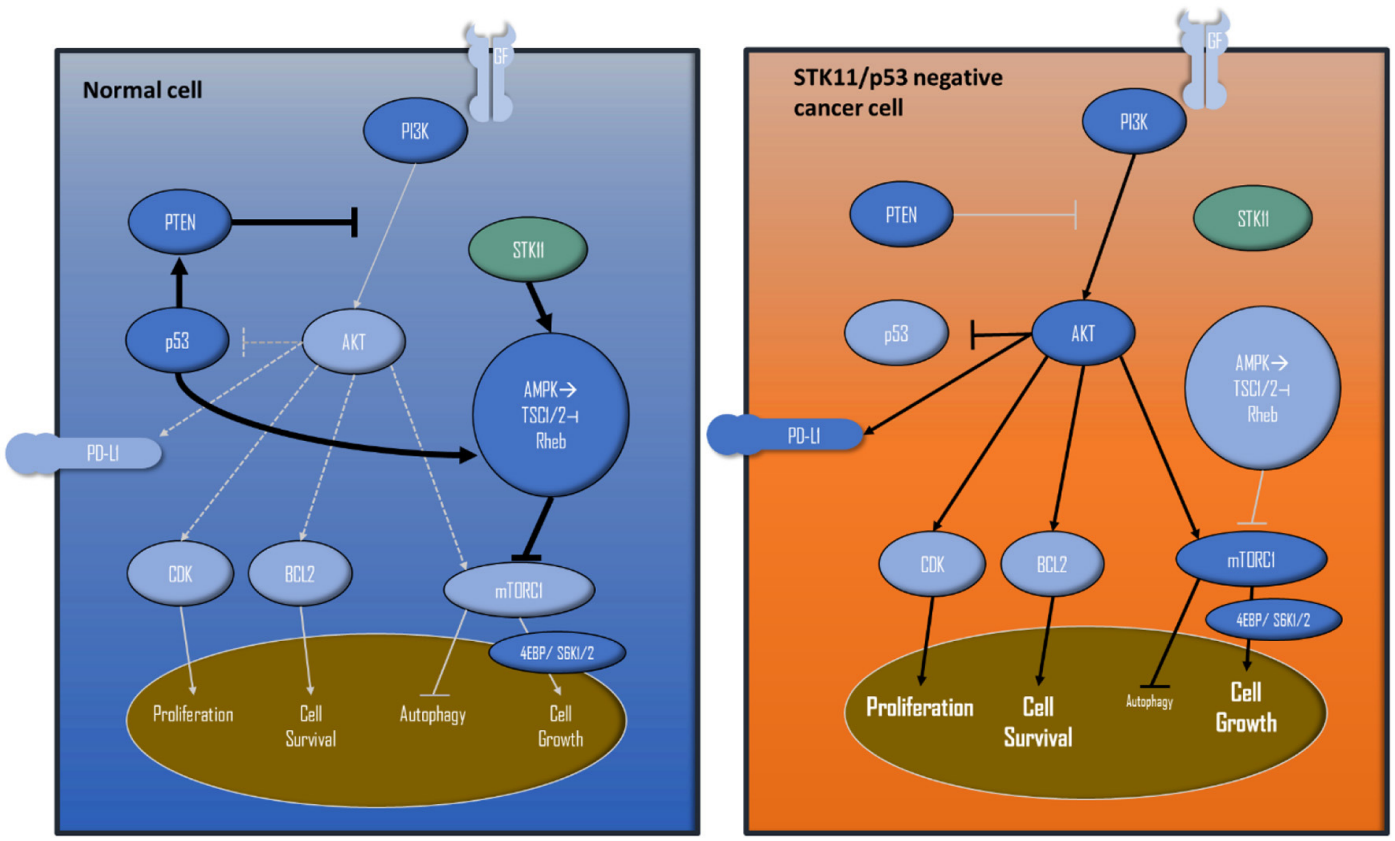
Simplified PIK3/AKT/mTOR pathway and interactions with STK11 and p53. The left shows a wild-type cell, the right cell displays how STK11 and p53 loss of function leads to extensive proliferation/cell survival and cell growth, because of the missing negative feedbacks and activations.

Since NGS revealed PIK3CA as the only targetable alteration and AKT together with transcription factors, such as BCL2 were upregulated, it appeared that the proliferation promoting influence was not triggered via the mTOR pathway. The therapy was focused on the PIK3CA mutation, again (Figure 3). We evaluated intrinsic resistance factors for PIK3CA inhibitors, no PTEN loss nor amplification of FGFR1 could be detected (25, 31).

The analysis of the PI3K pathway, including the peripheral effector components involved at the molecular and mRNA levels, indicated that the use of the PI3K inhibitor Alpelisib would not suffice to inhibit the complete PI3K/AKT pathway. This assumption is supported by the fact that important components of tumor metabolism like PEPCK (phosphoenol-pyruvate- carboxykinase), cell cycle progression like CDK20, Myc, and factors of cell survival like Mcl1, Bim were not upregulated. In addition, IRS family member 4, which constitutively hyperactivates the PI3K/AKT pathway, was downregulated on the mRNA level. Components of the cross-linked oncogenic pathway, such as Rat Sarcoma Virus (RAS), were not upregulated, therefore, inhibition of this pathway did not appear promising (32). To address this issue and to take into account the high proliferation rate, a cytostatic agent was administered in analogy to the study NCT04215003, together with Alpelisib.

For the first time, a remarkable therapeutic effect was observed. From ECOG II, the patient changed toward ECOG 0 within 3 weeks, also because oxygenation improved from 57 to 70 mm/Hg. Sonographically, the effusion was not traceable anymore. Seven weeks later she suffered from an etiologically unclear thoracic pain event and died.

The second patient presented herself with hepatic progress shortly after completing adjuvant therapies. Tumor profiling was performed based on a liver biopsy together with a blood sample. An STK11p.Y131^*^ mutation with clinical relevance was found. STK11/LKB1 mutations are reported in neuroendocrine tumors, such as large cell subtypes (33–35), but rare in breast cancers with an incidence of 0,2–1,0 % (35). STK11 alterations are associated with a lack of PDL1 expression, and the patient had a low TMB. Being MSI stable, no efficacy of checkpoint inhibitors was predicted (29).

The detected STK11mutation is considered to be a loss-of-function mutation resulting in activation of mTOR, as it is additionally induced by the detected p53 alteration (Figure 3). Functional loss of p53 activity can contribute to higher activities of the PI3K/AKT/mTOR pathways (36, 37).

To evaluate further the mTOR effect, we investigated additional peripheral effectors at the mRNA level.

Due to STK11 loss, the mTOR activation was most likely triggered *via* S6K1/2 (ribosomal S6 protein kinase 1/2), which was partially upregulated, stimulating proliferation by eIF4B and S6. Consequently, we applied the mTOR inhibitor, Everolimus, in this situation (35, 38–40).

Everolimus, itself, is approved by the Food and Drug Administration (FDA) for hormone receptor-positive and Her2 negative breast cancer. It is also the standard of care for NETs in National Comprehensive Cancer Network (NCCN) guidelines (41).

But mTOR inhibition as monotherapy based on allosteric inhibitors of mTORC1, like Everolimus, may lead to decreased therapeutic efficacy due to several resistance mechanisms: this could be incomplete inhibition of mTORC1, suppression of negative feedback loops, for example via increased IRS 1, which activates PI3K/AKT, ERK pathway activation, just to mention only a few of resistance factors (22, 25, 42). There is evidence of potential synergism with angiogenetic inhibitors. Taking into account the presence of upregulation of VEGFA and HIF-alpha-pathway, Bevacizumab was added to Everolimus (43–45).

Due to the highly proliferating disease and extent of metastasis, Capecitabine was administered in accordance with the test results (46). This is not surprising as Capecitabine is the standard of care to treat breast cancer, it is also mentioned in German guidelines for colorectal NETs or of NETs pancreatic origin. The therapy combination of Everolimus, Bevacizumab, and Capecitabine was well-tolerated. Imaging showed partial remission for 3 months. Then, the tumor progressed dramatically, and the patient died soon due to liver insufficiency.

## Conclusion

To date, molecular profiling is used especially in breast, lung, colorectal, prostate, and gastric cancer (47). Here we demonstrate two patients with a rare tumor entity as a role models to illustrate the benefit to which a broader molecular tumor profiling can offer a significant contribution not only to diagnosis but also to the therapeutic regime.

Therapy-relevant mutations were uncovered, analyzing numerous tumor-relevant genes (>400) and pharmacogenomics. Specifically, the intelligent combination of immunocytochemistry/-histochemistry, chemosensitivity testing on tumor cells, DNA alterations, and expression profiles, could be detected and delivered valuable insights to tailor therapy.

Hence the rate of ineffective and cost-intensive therapies can be diminished and will improve the already available personalized, targeted therapies. Currently, application of solitary genetic testing delivers advantages only to a minority of patients (48–50). The first basket trials especially like the SHIVA trial largely failed because molecular filters were applied (48). Newer trials like the RESILIENT trial had beneficial outcomes even in late-stage patients with several previous therapies applying enhanced molecular analysis comprising also cytological features and other cancer characteristics (15).

Promising new options are especially needed for rare tumor entities, exemplified by NEBC, which remains a major diagnostic and therapeutic challenge today. From the start, there are numerous pitfalls in diagnosing NEBC, because it is itself a heterogeneous group of tumors. The rarity of this tumor type makes it imperative to apply sensitive diagnostic tools for effective treatment options.

It is also important for patients, for whom empirical therapy showed no efficiency and potential therapies based on tumor-specific profiles, respecting possible resistance mechanisms are explored. Viewed in isolation, not only the targets may be considered for the choice of therapy, but, if possible, the context of the whole pathway network together with other biomarkers too.

Questions that we need to ask, is whether the therapeutic effect justified the application of comprehensive tools in these cases. In both heavily pre-treated patients, actionable targets were discovered together with findings from ICC, chemosensitivity, and pathway modeling leading to a treatment that was well-tolerated and an improvement in the overall situation. However, both patients suffered from a highly aggressive subtype and were already in the metastatic situation where curative treatment is virtually not possible and the effects of the treatment did not last longer than a few months. Especially in rare cancers, where the prognosis is worse from the start, we should think about using tailored therapies based on comprehensive tumor characteristics earlier, because, only then, can we know whether this approach will provide a benefit. Trials to combine several rare cancer types and extensive profiling could hold the key to a successful treatment.

## Data Availability Statement

The datasets presented in this article are not readily available because of ethical and privacy restrictions. Requests to access (no. E-MTAB-11703) the datasets should be directed to the corresponding author.

## Ethics Statement

Ethical review and approval was not required for the study on human participants in accordance with the local legislation and institutional requirements. The patients/participants provided their written informed consent to participate in this study.

## Author Contributions

DS-N: counseling of patients, clinical management, data analysis, data interpretation, and manuscript writing. RV: counseling of patients, data interpretation, and manuscript review. AT: data interpretation and manuscript review. SS: data analysis and manuscript writing. All authors contributed to the article and approved the submitted version.

## Conflict of Interest

SS is employed by Datar Cancer Genetics Europe GmbH. The remaining authors declare that the research was conducted in the absence of any commercial or financial relationships that could be construed as a potential conflict of interest.

## Publisher's Note

All claims expressed in this article are solely those of the authors and do not necessarily represent those of their affiliated organizations, or those of the publisher, the editors and the reviewers. Any product that may be evaluated in this article, or claim that may be made by its manufacturer, is not guaranteed or endorsed by the publisher.
